# Access to Diagnostic Tests and Essential Medicines for Cardiovascular Diseases and Diabetes Care: Cost, Availability and Affordability in the West Region of Cameroon

**DOI:** 10.1371/journal.pone.0111812

**Published:** 2014-11-04

**Authors:** Ahmadou M. Jingi, Jean Jacques N. Noubiap, Arnold Ewane Onana, Jobert Richie N. Nansseu, Binhuan Wang, Samuel Kingue, André Pascal Kengne

**Affiliations:** 1 Department of Internal Medicine and Specialties, Faculty of Medicine and Biomedical Sciences, University of Yaoundé I, Yaoundé, Cameroon; 2 Internal Medicine Unit, Edéa Regional Hospital, Edéa, Cameroon; 3 National Obesity Center, Yaoundé Central Hospital, Yaoundé, Cameroon; 4 Sickle Cell Unit, Mother and Child Centre, Chantal Biya Foundation, Yaoundé, Cameroon; 5 Department of Population Health, Division of Biostatistics, New York School of Medicine, New York, United States of America; 6 Non-communicable Disease Research Unit, South African Medical Research Council and University of Cape Town, Cape Town, South Africa; University of Perugia, Italy

## Abstract

**Objective:**

To assess the availability and affordability of medicines and routine tests for cardiovascular disease (CVD) and diabetes in the West region of Cameroon, a low-income setting.

**Methods:**

A survey was conducted on the availability and cost of twelve routine tests and twenty medicines for CVD and diabetes in eight health districts (four urban and four rural) covering over 60% of the population of the region (1.8 million). We analyzed the percentage of tests and medicines available, the median price against the international reference price (median price ratio) for the medicines, and affordability in terms of the number of days’ wages it would cost the lowest-paid unskilled government worker for initial investigation tests and procurement for one month of treatment.

**Results:**

The availability of tests varied between 10% for the ECG to 100% for the fasting blood sugar. The average cost for the initial investigation using the minimum tests cost 29.76 days’ wages. The availability of medicines varied from 36.4% to 59.1% in urban and from 9.1% to 50% in rural settings. Only metformin and benzathine-benzylpenicilline had a median price ratio of ≤1.5, with statins being largely unaffordable (at least 30.51 days’ wages). One month of combination treatment for coronary heart disease costs at least 40.87 days’ wages.

**Conclusion:**

The investigation and management of patients with medium-to-high cardiovascular risk remains largely unavailable and unaffordable in this setting. An effective non-communicable disease program should lay emphasis on primary prevention, and improve affordable access to essential medicines in public outlets.

## Introduction

Hypertension, diabetes and consequential cardiovascular diseases (CVD), have reached epidemic proportions worldwide, and disproportionately affect low- and middle-income countries (LMICs) [1]. Nearly 80% of deaths from CVD and diabetes occur in LMICs, resulting in non-communicable diseases (NCDs) overtaking communicable diseases as the major leading cause of disease and death in LMICs [1]. Moreover, the economic and societal impact of CVD on individuals, households and countries is enormous. These effects are particularly important in LMICs, where CVD more often affects individuals in their working age, and therefore contributes disproportionately to losses of potential years of healthy and active life, which can impair economic growth [2]–[4]. Indeed, it has been demonstrated that through their combined impact on economies, health systems, households and families, diabetes and CVD are major threats to the current and future economic development and prosperity of individuals and societies in Africa [5].

Timely interventions including early detection, lifestyle changes and use of effective and affordable medicines, have been shown to reduce morbidity and mortality associated to CVD [6]. Unfortunately, studies have shown that these essential interventions for CVD and diabetes are not always available and accessible in the public sector in LMICs [7], [8]. Although there is a better availability of these interventions including medicines for CVD and diabetes in the private sector, the end-user cost is a major deterrent to access to the large segment of the population in LMICs. Moreover, because prevention and treatment interventions for CVD and diabetes are mostly purchased through out-of-pocket payments in LMIC, treatment for NCDs is largely unaffordable in these settings [4], [9].

In Cameroon where more than 50% of the population live below the poverty line and where there is no effective health insurance system [10], [11], out-of-pocket payments represented 94.5% of private health expenditures in 2011 [12]. CVD and diabetes are growing public health concerns in Cameroon [13]. Major deficiencies exist in both the quality and access to care such that these NCDs and their risk factors are diagnosed infrequently and managed inadequately. Interestingly however, CVD and diabetes are increasingly receiving more from the Cameroon government [13]. Since 2001, a number of health policies on NCDs including CVD and diabetes have been formulated and adopted by the National Ministry of Public Health. The recognition of hypertension and diabetes as emerging public health threats has led to the creation of the National Diabetes-Hypertension Control Program which aims to promote equitable access to quality health services in order to reduce the morbidity and mortality from these conditions [14]. To achieve this goal, making essential medicines for CVD and diabetes available and affordable is paramount. Therefore, data on the current access to CVD and diabetes care are crucial for shaping and improving strategies to combat CVD and diabetes.

In May 2003, the World Health Organization (WHO) in collaboration with Health Action International developed a standardized method for surveying medicine prices, availability, affordability, and price components in LMIC [15]. Using this standardized method, the present study aimed to assess the prices, availability and affordability of selected routine tests and common essential medicines for CVD and diabetes care in Cameroon.

## Methods

### Ethics statement

The study was approved by the Regional Office of the Ministry of Public Health (MOH) for the West region, acting as Ethics Committee. Signed authorization was obtained from the head of each participating outlet, who also identified the relevant informant. All informants interviewed in the study provided a verbal consent to take part in the study. Verbal consent was approved by the Ethics Committee and found to be appropriate since this study did not involve data collection on individual subject. The consent was documented by a tick of boxes in our data collection log book. Each of them was individually informed on the intended use of the data collected and the confidentially surrounding the exploitation of the data. They were further advised that they were under no obligation to take part in the study, and that by accepting to respond to our questionnaire they were consenting to take part in the study and allowing the collected data to serve the intended purpose.

### Setting

We conducted a survey in the West Region of Cameroon which, had in 2010 a population of 1,785,285 inhabitants (9.2% of the country’s total population), with a density of 128.5 inhabitants/km^2^ (2^nd^ most densely populated region) [16]. The region is divided into 20 health districts, and has a total of 530 health facilities, both public and private. Public outlets acquire medicines from the state owned Regional Centre for the Essential Drug Program and sell them to the public on a cost-recovery basis following the national guidelines. Private outlets acquire medicines from a private accredited supplier, LABOREX and sell them to the public at prices different from those recommended by the national guidelines.

### Selection of medicine outlets

Facilities were selected based on study feasibility, whether urban or rural and the size of the population served, with the aim of sufficiently representing the region. We selected 6 urban and 5 rural outlets. The main hospital of the capital of the region was included, as well as one pharmacy selected as the private outlet. This pharmacy is a popular medicine outlet with the highest anticipated chance of getting all the needed medicines. It served as the reference medicine outlet. One market vendor was also sampled in the regional capital main market to serve as a reference outlet in the informal circuit. The remaining 3 urban and 5 rural outlets selected were those reachable within one-day’s drive from the capital; they were all public outlets (state administered), district hospitals or a sub-divisional hospital. In total, there were 2 private outlets (one in the formal sector and one in the informal circuit), and 9 public outlets. In all, the selected sites served over 60% of the population of the region, distributed in eight health districts, four rural and four urban (Table 1).

**Table 1:** Availability of medicines and test per site and the population served.

Survey site	Area	Population served2012 estimates	Availability ofMedicines	Availability of WHOrecommended tests	Availability ofall tests
Foumban	Urban	170,571	36.4%	83.3%	91.7%
Dschang	Urban	211,818	45.5%	100%	100.0%
Mifi	Urban	254,123	59.1%	66.7%	58.3%
Foumbot	Rural	59,223	45.5%	66.7%	50.0%
Bangourain	Rural	29,694	50.0%	33.3%	25.0%
Balessing^a^	Rural	NA	18.2%	50.0%	41.7%
Penka Michel	Rural	88,338	22.7%	83.3%	75.0%
Mbouda	Urban	250,967	50.0%	83.3%	83.3%
Batcham	Rural	90,271	9.1%	50.0%	41.7%
Salvia^b^	Urban	NA	100%	16.7%	NA
Market^b^	Urban	NA	63.6%	NA	NA

NA: Not Applicable.

a Found in Penka-Michel health district.

b Found in the Mifi health district.

### Selection of medicines and diagnostic tests to be surveyed

The survey collected information on 20 medicines used to treat CVD and diabetes. Medicines were selected in line with a previous work by a WHO team on selected essential medicines for chronic diseases in six low and middle-income countries [7], their potential impact on the burden of disease and their availability in standard formulations. The majority of these medicines are included in the WHO model list of essential medicines [17]. Diagnostic tests for CVD and diabetes included the minimal WHO workup, and other tests that were considered relevant for assessment and management of cardiovascular risk as recommended by the WHO [18]. The selection was finalized after consultation with local experts.

### Data collection

Data were collected by a single trained investigator in all the sites during a one week period in 2012, using a pre-conceived questionnaire, based on the standardized method for surveying medicine prices, availability, affordability, and price components in LMICs developed by the WHO and Health Action International [15]. For each medicine and at each outlet, information included the availability of any drug dosage of the cheapest form (originator brand or generic) and the cost per tablet or vial paid by clients. The price collected from each outlet was the price charged directly to clients. Data on tests were equally collected on the same checklist. These data on tests were collected in the hospitals surveyed. In the absence of social security system in Cameroon, medications and tests covered in the current study are paid for essentially from out-of-pocket money.

### Data analysis

This was a pilot study based on a convenient sample, and accordingly no formal power estimation. We assessed the availability of medicines in two ways: 1) by estimating the percentage (%) of the 20 medicines available in each site and 2) by estimating the percentage (%) of outlets in which any dose or form of the medicine was found. Availability less than 30% is considered very low and greater than 80% is considered high [19]. We similarly determined the availability of tests as the percentage of outlets in which a test was found and the percentage of tests found at each site. We also estimated the median price ratio (MPR) of each medicine. This ratio compares a medicine’s median local price to its international reference price (IRP) in the Management Sciences for Health (MSH) 2012 Price Indicator Guide [20]. MSH international reference prices, which are generally offered by both not-for-profit and for-profit suppliers to developing countries, are recommended as the most useful standard. When no supplier prices were available, buyer prices were used. The MPR is used to express how much greater or less the median local medicine price is than the IRP. For example, an MPR of 2 would mean that the local medicine price is twice the IRP. Generally, a MPR of one or less indicates an efficient public sector procurement system. For patients’ medicine prices, MPR ≤1.5 were considered reasonable for all medicine forms (usually higher for innovator medicines) [7].

Affordability was estimated using the median medicine prices of public medicine outlets, or private outlet when this was not available in the public outlet, and the salary of the lowest-paid unskilled government worker which was 28,000 FCFA/month (USD 53) at the time of the survey, and calculating the number of days’ wages required to pay for investigation tests and to purchase a one-month course of treatment. Treatments costing one day’s wage or less (for a 30-day supply of medicine) were considered affordable.

## Results

### Availability of tests and medicines

The profile of the survey sites is described in Table 1. The availability of investigation tests is summarized in Table 2; ranging from 10% for the ECG to 100% for fasting blood sugar. It was markedly higher in the urban setting. Across study sites, the availability of investigation tests ranged from 58.3% to 100% for urban sites and 41.7% to 75% for rural sites (Table 1).

**Table 2:** Availability, affordability and median price of selected tests in the investigation of cardiovascular disease and diabetes.

Test	Availability			Median price		Number of days' wage for investigation
	Rural	Urban	All sites	Local (FCFA)	US Dollar	
Glycaemia	100%	100%	100%	1,250	2.38	1.34
Creatinemia	80%	100%	80%	3,000	5.72	3.21
Urea	80%	100%	80%	3,000	5.72	3.21
Total cholesterol	20%	75%	40%	3,350	6.39	3.59
HDL cholesterol	20%	75%	40%	3,350	6.39	3.59
Triglyceride	20%	75%	40%	2,850	5.43	3.05
Proteinuria	100%	100%	90%	1,000	1.91	1.07
Kaliemia	40%	100%	60%	3,000	5.72	3.21
HbA1c	0%	50%	20%	11,750	22.40	12.58
Uric acid	0%	100%	40%	3,100	5.91	3.32
Full blood count	80%	100%	80%	3,750	7.15	4.02
ECG	0%	25%	10%	10,000	19.07	10.7

Tables 1 and 3 show the availability of medicines used to treat cardiovascular disease and diabetes. Only 9 medicines surveyed had availability greater than 50%. This was higher in urban settings except for propranolol and Methyl-penicillin which was higher in the rural settings (Table 3). Up to 7 of the surveyed medicines were found only in private medicine outlets. Medicines’ availability across public pharmacies in different sites ranged between 36.4% and 59.1% in urban sites and between 9.1% and 50% in rural sites (Table 1). Medicines’ availability was higher in the urban informal sector where up to 63.6% of the medicines were available.

**Table 3:** Availability, affordability, median price and ratios of Medicines.

Drug presentation	Availability				Median price (per tablet or vial)				Defineddaily dose	Cost of Dailytreatment (USD)	Cost of 30 days'treatment (USD)	Number of days'wage for 1 month
	Rural	Urban	Reference(Pharmacy)	All sites	Local price(FCFA)	Local price(USD)	WHO price(USD)	Ratio*				
Acetylsalicylic acid 500 mg	40%	100%	100%	70%	5.0	0.01	0.0045	2.22	100 mg	0.002	0.06	0.03
Atenolol 50 mg	0%	0%	100%	10%	78.1	0.15	0.0106	14.15	75 mg	0.23	6.75	3.92
Benzathine-benzylpenicilline2.4MIU	100%	100%	100%	100%	210.0	0.40	0.3241	1.23	2MIU	0.40	12.00	6.74
Captopril 25 mg	0%	50%	100%	30%	100.0	0.19	0.0216	9.80	50 mg	0.38	11.40	6.41
Ramipril 5 mg	0%	25%	100%	20%	240.1	0.46	NA	NA	5 mg	0.84	25.20	14.16
Hydrochlorothiazide 50 mg	20%	50%	100%	40%	10.0	0.02	0.0050	4.00	25 mg	0.01	0.30	0.17
Furosemide 40 mg	100%	100%	100%	100%	5.0	0.01	0.0061	1.64	40 mg	0.01	0.30	0.17
Isosorbide dinitrate 5 mg	0%	0%	100%	10%	81.6	0.16	0.0142	11.27	20 mg	0.64	19.20	10.79
Losartan 50 mg	0%	0%	100%	10%	685.4	1.31	0.0185	70.81	50 mg	0.76	22.80	12.81
Simvastatin 20 mg	0%	0%	100%	10%	295.0	0.56	0.0252	22.22	20 mg	1.81	54.30	30.51
Methyldopa 250 mg	40%	75%	100%	60%	30.0	0.06	0.0295	2.03	1 g	0.24	7.20	4.10
Nifedipine 10 mg	20%	75%	100%	10%	20.0	0.04	0.0130	3.08	30 mg	0.12	3.60	2.02
Nifedipine 20 mg SR	0%	25%	100%	20%	42.3	0.08	0.0233	3.43	30 mg	0.12	3.60	2.02
Phenoxymethylpenincilline250 mg	60%	50%	100%	60%	15.0	0.03	0.0158	1.90	3MIU	0.18	5.40	3.03
Propranolol 40 mg	20%	0%	100%	20%	26.9	0.05	0.0059	8.47	160 mg	0.20	6.00	3.37
Spironolactone 50 mg	0%	0%	100%	10%	156.7	0.30	0.0338	8.87	75 mg	0.45	13.50	7.58
Heparine 5000 UI	0%	0%	100%	10%	2 835.0	5.41	0.9055	5.97	10000 IU	10.82	324.60	182.36
Glibenclamide 5 mg	60%	100%	100%	80%	5.0	0.01	0.0042	2.38	10 mg	0.02	0.60	0.34
Metformine 500 mg	60%	100%	100%	80%	7.5	0.01	0.0168	0.59	2 g	0.04	1.20	0.67
Actrapid 100 IU/ml	60%	100%	100%	80%	3,000.0	5.72	0.7723	7.41	40 IU	0.23	6.86	3.85
Insulatard 100 IU/ml	0%	0%	100%	10%	3,000.0	5.72	0.7723	7.41	40 IU	0.23	6.86	3.85
Mixtard 100 IU/ml	60%	100%	100%	80%	14,500.0	27.65	0.6143	45.01	40 IU	1.11	33.30	18.70

*Ratio: Local price/WHO price.

NA: Data not available in the 2012 edition.

### Median prices and affordability of tests and medicines

The affordability of investigation tests and treatment was estimated as the number of days’ wages the lowest-paid government worker would be required to pay for the initial tests and one-month course of treatment from the public sector, and when this is not available, from the private sector. This is summarized in Tables 2 and 3. Of all the medicines surveyed, only the oral antidiabetics, hydrochlorothiazide and acetylsalicylic acid cost less than 1 day’s wage. Statins remained largely unaffordable. Only metformin and benzathine-benzylpenicilline had an acceptable median price ratio of 0.59 and 1.23 respectively.

Table 4 depicts the minimum estimated cost of management and monitoring of different CVD risk profiles. The minimum number of tests to investigate a newly diagnosed hypertensive patient including a fasting blood sugar, cholesterol, potassium levels, proteinuria and ECG cost 29.76 days’ wages. Risk monitoring which comprises lipid profile, fasting blood sugar and proteinuria cost 12.6 days’ wages. As this cumulates over the months, 12-monthly monitoring and 6-monthly monitoring cost 1.05 days’ wages and 2.11 days’ wages. Treating a patient with ischemic heart disease with a drug combination comprising a β-blocker (atenolol), Angiotensin Converting Enzyme Inhibitor (captopril), statin (simvastatin) and acetylsalicylic acid will cost at least 41.33 days’ wage. This was largely unaffordable.

**Table 4:** Estimated costs of managing cardiovascular risk profile.

Purpose		Components	Cost of tests(days’ wages)	Costs of drugs(days’ wages)	Total cost(days’ wages)
Diagnosis and stratification	Absoluterisk	Minimal WHO recommended tests	29.76	NA	29.76
Primaryprevention	<10% Risk	Lifestyle changes and 12-monthly risk monitoring	1.05	NA	1.05
	10−20%Risk	Lifestyle changes and 6-monthly risk monitoring	2.11	NA	2.11
	20−30%Risk	Statin/Antihypertensive/6-monthly risk monitoring	2.11	30.67	32.78
	≥30%Risk	Statin/Antihypertensive/Acetylsalicylic acid/3-monthlyrisk monitoring	4.22	30.70	34.92
Secondaryprevention		β-Blocker, ACEI, Statin, Acetylsalicylic acid/3-monthlyrisk monitoring	4.22	37.11	41.33

Minimal WHO recommended tests include fasting blood sugar, cholesterol, potassium levels, proteinuria and ECG.

Risk monitoring includes lipid profile, fasting blood sugar and proteinuria. It cost 12.6 days’ wages. As this cumulates over the months, 12-monthly monitoring and 6-monthly monitoring cost 1.05 days’ wages and 2.11 days’ wages.

NA: not applicable.

WHO: World Health Organization; ACEI: Angiotensin Converting Enzyme Inhibitor.

### Comprehensive analysis of medicine availability and price

**Figure 1:**
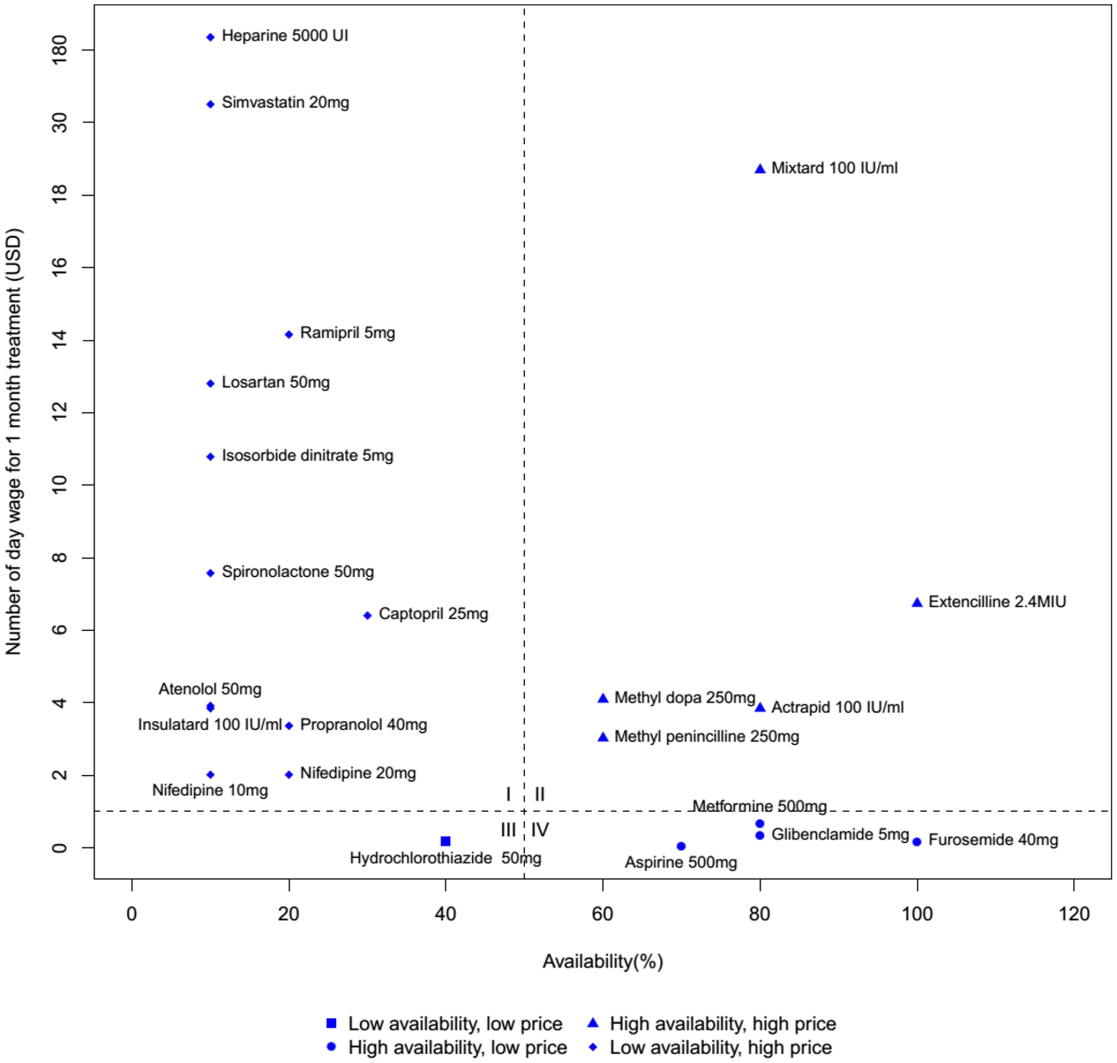
Comprehensive analysis of medicine availability and affordability. The availability (%) each drug is depicted on the x-axis, while the y-axis shows the price (days’ wages).

Figure 1 presents the availability and price of medicines surveyed. The availability score for each medicine is depicted on the x-axis, while the y-axis shows the value of the number of days’ wage required to purchase a one-month course of treatment. The figure can be divided roughly into four quadrants. The upper left quadrant (quadrant I) contains drugs with high number of days’ wage and low availability. In the case of these medicines, heparine and simvastatin for instance, patients face both high costs and high difficulty in obtaining them.

The lower right quadrant (quadrant IV) contains drugs with low number of days’ wage and high availability. Furosemide had the highest availability (100%) and one of the lowest number of days’ wage (0.17). Acetylsalicylic acid was the most affordable medicine, with a 0.03 day’s wage, and had an availability of 70%.

## Discussion

This survey is the first of this nature in Cameroon and in a large number of low-income countries using standard methods developed and validated by the WHO [15], and with emphasis on two common NCDs in LMICs. We focused on what patients pay for available tests and medicines at the end of the spectrum and thus, did not look at markups, taxes, other distribution costs and different drug forms or conditionings, which explained differences in prices observed in surveys carried out in geographically diverse sites [7], [8]. We carried out the survey in a geographically limited site. However, key aspects of availability and affordability of essential medicines are similar to other studies and are thus, comparable.

The investigation and management of patients with medium-to-high cardiovascular risk remains largely unavailable and unaffordable in this low income setting. The minimum recommended tests by the WHO (blood sugar, potassium, creatinemia and proteinuria) had a satisfactory availability of 80% or more, and comparably between rural and urban settings. Lower availabilities of these tests have been found in Benin and Sudan [21]. Lipid profile had low availability, especially in rural settings, and ECG which was seen only in few urban facilities was largely unavailable. HbA1c, a key-test in the monitory of diabetic patients also had very low availability, and was very unaffordable. Only two tests (blood sugar and proteinuria) cost less than 2 days’ wages and could therefore be considered affordable. Overall, the cost of minimal WHO recommended tests for initial diagnosis and cardiovascular risk stratification was very unaffordable, representing one month salary for the lowest-paid unskilled government worker.

The availability of medicines in our study, although higher than that reported in six LMICs [7], was not always optimal, therefore indicating the existing scope of improvement. The lessons learned from the HIV/antiretroviral experience could be applied in order to improve access to essential medicines for NCDs [22], [23]. A recent study evaluating the preparedness of a representative sample of 24 public and not-for-profit Tanzanian health facilities to provide routine care for hypertension and diabetes, has shown that compared with care for HIV, health facilities at all levels were ill-prepared to address the diagnosis, treatment, and ongoing monitoring needs of patients with NCDs. These results suggest that primary-care system for HIV could serve as both model and foundation to improve CVDs and diabetes care including access to essential medicines [24]. Practically, to improve the availability of essentials medicines for CVDs and diabetes in LMICs like Cameroon, ministries of health should optimize the local procurement practices and organize an efficient supply chain all over the country [22]. To assure a continuous availability of these medicines, good monitoring systems for stock levels and use are crucial. The use of wireless communication technologies, such as short message service (SMS), has been shown to increase procurement efficiency and monitoring. Community volunteers can track the availability of medicines in local facilities, and their SMS messages can feed into a website that helps to identify underperforming districts and start corrective action [22], [23]. Similar approaches for first-line antimalarial drugs have resulted in a 50% decrease in facility stock-outs in a pilot project to improve drug supply management in rural Tanzania [25].

Despite the relatively good availability of the surveyed medicines, affordability was also very low with only six of the surveyed medicines that cost less than a day’s wage. In a similar recent study in China, these costs were much lower than those found in our study: 0.1 days’ wages for captopril 25 mg, 0.3 days’ wages for metformine 500 mg, 1.4 days’ wages for nifedipine 20 mg and 6.6 days’ wages for losartan 50 mg [26]. A 1-month course of combination therapy for the secondary prevention of coronary heart disease cost 40.87 days’ wages in our setting compared to 18 days’ wages in Malawi and 1.5 days’ wages in Sri Lanka [7]. The unaffordability of most of medicines would be majored if additional costs of consultation and diagnostic tests were accounted for. A study among Cameroonian informal sector workers which represent the vast majority of the active population in the country, found that only 4.4% of them had health insurance [11]. It has been shown that catastrophic payments and impoverishment due to cardiovascular diseases are more common in people with no health insurance than in those with health insurance [27]. Scaling up the health insurance coverage of the Cameroonian population is therefore crucial to improve access to CVD and diabetes care. Generally, medicines on the national essential medicines list (NEML) are the highest priority for coverage by the health insurance program in sub-Saharan Africa [22]. In the national health insurance system in Rwanda, for example, members are eligible to receive NEML drugs for outpatient treatment with a 10% copayment [28].

Policy options to improve access to affordable medicines through cost containment and promotion of generics have been suggested. Some which could be implemented in the Cameroonian context include: involvement of reputed organizations and of patients associations and consumers groups to raise awareness on the benefit of good quality generics; reliable quality assurance capacity to demonstrate the bioequivalence between generics and originator brands; encouraging generic prescribing and influencing the patient to request for prescription of cheaper version of medicines [29]–[31]. Generic substitution could lead to significant financial savings. In France for instance, implementation of a general generic substitution strategy saved nearly US$2 billion in 2008 alone [32]. Nonetheless, assurance of generic medicine quality is crucial to protect the population from potentially harmful effects or inefficient medicines and reduce waste of resources. Because of inadequate regulation and insufficient penalization, substandard medicines are common in developing countries [23]. In Rwanda, 20% of hypertensive medicines purchased on the market were of substandard content and 70% were of insufficient stability [33].

Prices of medicines could be reduced by improving purchasing efficiency, eliminating taxes and regulating markups. The analysis of the MPR of the surveyed medicines shows that the local procurement and retail system was inefficient, with most of the MPR greater than 1.5. MPR was acceptable only for metformin and benzathine-benzylpenicilline, and was as high as 70.81 for losartan. Comparatively, a recent study in New Delhi, India, has shown that procurement prices of surveyed medicines were reasonable [34].

The present study has some limitations. First, sampling of survey sites was not random with a risk of sampling bias. However, for equity, strength and feasibility, we surveyed 8 health districts, urban and rural, that served the majority of the population of the region. Second, data on availability were collected at a particular point in time and thus, they do not reflect fluctuations in the availability over time. Third, our study present limited data from the private sector. However, as medicines are usually cheaper in the public sector [7], our study which mainly focuses on data from the public sector present the “best case scenario” of the affordability of drugs surveyed.

## Conclusion

This study has provided some insight on issues relating to access to cardiovascular and diabetes care in Cameroon. The investigation and management of patients with medium-to-high cardiovascular risk remains largely unavailable and unaffordable in this low income setting. An effective NCD program should lay emphasis on primary prevention, and improve access to affordable generic essential medicines in public outlets.
